# Bacteria

**Published:** 1883-01

**Authors:** L. S. Beale


### BACTERIA.

Time will probably show that many of the pretentious statements which have been made concerning the fungous germ hypothesis of contagious diseases are groundless.

There is, probably, not a part of the body of any one of us a quarter of an inch in diameter where bacteria germs are not present. Certainly every time we eat, myriads are carried into our alimentary canal ; and every time we breathe, except in the very purest atmosphere, multitudes pass into the air-passages. So small are these bacterial germs that they would pass without the slightest difficulty through basement membrane and through the interstices of any of the tissues of the organism ; and yet the public is taught that there is some intimate connection between bacteria and dust and morbid phenomena. Erroneous notions are spread far and wide by sensation lectures, under such a title as "Dust and Disease." The dust which causes disease is of a most exceptional kind. It has been said that the air of the Swiss mountains is devoid of bacteria. But is the health and vigor of the inhabitants of the Alps to be compared with that of the workers on the Paddington Dust Heaps? As a fact, ordinary bacteria are harmless enough ; they exist in us without disturbing us in any way, but they only grow and multiply in great numbers when circumstances become favorable. I can give you positive proof that bacteria germs exist not only upon the surface of the skin and mucous membranes, but in the internal organs, in the interstices of healthy tissues and in the blood itself. Some years ago I examined the layers of a fibrinous clot which had been slowly formed from the blood in the interior of a large aneurism of the aorta of a man who died of the disease. The body was examined six or eight hours after death. The aneurism had existed for many years ; and probably some of the layers of fibrin which had been deposited were almost as old as the aneurism itself. Now, I found that in all parts of the firm, laminated, leather-like material, which served to greatly increase the wall of the aneurismal sac, there were indications of disintegrating changes having taken place. Upon carefully examining minute pieces of the fibrin under high powers, multitudes of bacteria and their germs were discovered without difficulty. But the older layers in the outer part were here and there softened, and portions of the fibrinous matter seemed eroded, many small masses of soft and broken-down material being present. All these teemed with bacteria, moving, growing and multiplying.

Now, these bacteria, like the fibrin in which they were growing and multiplying, were close to the blood, and within the vascular system; internal to the various tissues constituting the wall of the vessel, which was dilated to form the aneurismal sac. The bacteria must have been growing and multiplying in the lifetime of the patient, and probably for many months before his death occurred. They could not have got into the position in which they were discovered from the outside, for it is hardly conceivable that such an organism as a bacterium could have found out, while outside the body, that within the vascular system there was material suitable for its growth and multiplication. Neither is it possible that the bacteria could have made their way from without to the situation in which they were found, nor could they have effected, in the course of a few hours, the extensive erosions and softening discovered. Such theories could not be sustained with any show of reason. The only conculsion, therefore, which is in accordance with the facts in the case and with common sense, is that which I have before adverted to, viz., that bacteria germs exist at all times in all parts of the body, even in the blood itself, during the healthy state.

I conclude that as long as the normal state of things exists the living bacteria germs in all parts of the organism do not grow and multiply, but when any change occurs of the character of that which results in chemical decomposition, these bacteria germs multiply. This multiplication proceeds, although we are alive, just as it takes place in dead animal and vegetable matter. And it will occur in every part of every one of us a very few hours after death.

So you see that if bacteria germs constitute the actual, material, living particles by which contagious disease is propagated, they must be peculiar bacteria, totally different from the ordinary bacteria germs which exist and have existed everywhere. The ordinary bacteria may certainly grow and multiply enormously on the mucous membranes of the body, in follicles of the mucous surfaces and in viscera—intestinal canal, bladder, and passages therefrom, nay, even among the elements of healthy growing tissue, without causing any disease at all.

Bacteria germs, low fungi and algæ exist in connection with the tissues and fluids of every human organism, and, as you may convince yourselves at any time, millions of these are unquestionably present during every moment of existence in health on the dorsum of the tongue. Multitudes, as I have said, pass down the alimentary canal every time we swallow food or fluid. Such ordinary bacteria and their germs do us no harm whatever. But please do not infer from what I have said that putrid fluids loaded with bacteria are inocuous or to be recommended. Organic matter in a state of putrefactive decomposition, when introduced into the alimentary canal, gives rise to pathological phenomena irrespective of the bacteria it may contain.—*L. S. Beale, M. B., F. R. S., in Slight Ailments.*

